# Lifestyle Behavior Patterns During the Transition From Adolescence to Emerging Adulthood: Associations With Mental Health and Wellbeing

**DOI:** 10.1177/21676968251376750

**Published:** 2025-09-06

**Authors:** Matthew Bourke, Denver Brown, Matthew Y.W. Kwan

**Affiliations:** 1 The Health and Wellbeing Centre for Research Innovation, School of Human Movement and Nutrition Sciences, 1974 The University of Queensland, Brisbane, QLD, Australia; 2 Department of Kinesiology, Kansas State University, Manhattan, KS, USA; 3 Department of Child and Youth Studies, Brock University, St. Catherines, ON, Canada; 4 Department of Family Medicine, Infant Child and Health Lab, McMaster University, Hamilton, ON, Canada

**Keywords:** physical activity, screen time, sleep, risky behaviors, distress, wellbeing

## Abstract

The synergistic role played by multiple lifestyle behaviors on mental health during the transition from adolescence to emerging adulthood has not been extensively studied. This study included 493 participants who self-reported on a range of health behaviors during adolescence and emerging adulthood, and psychological distress and mental wellbeing during emerging adulthood. Latent profile analysis and latent transition analysis were used to analyze the data. Three unique behavioral profiles were observed at baseline: moderately physically active abstainers (i.e., abstaining from alcohol, tobacco, and marijuana), high screen time users, and moderately physically active and moderate risk behaviors. Four unique behavioral profiles were observed at follow-up: highly physically active with moderate risk behaviors, moderately physically active with moderate risk behaviors, physically inactive abstainers, and physically inactive with risky behaviors. Adolescents characterized as moderately active abstainers reported fewer symptoms of psychological distress during emerging adulthood compared to adolescents who displayed moderately active and moderate risk behaviors, and better mental wellbeing than high screen time users.

## Introduction

The transition from adolescence to emerging adulthood is a critical time in life which can have a profound and long-lasting impact on an individual’s future health and wellbeing (Bonnie et al., 2014). This is a period for which young people establish independence, assume new responsibilities and obligations, and adopt long lasting (un)healthy patterns of behaviours beginning in young adulthood (Kwan et al., 2012; Nelson et al., 2008; Sawyer et al., 2012). Correspondingly, this transition period is characterized by substantial physical, affective, cognitive, and behavioural changes, which may leave young people susceptible to experiencing poorer mental health (Paus et al., 2008; Steinberg, 2005). For example, the burden of common mental health disorders substantially increases during adolescence and the burden of mental health disorders reaches a peak in emerging adulthood (Solmi et al., 2022; Whiteford et al., 2013) and it is estimated that approximately one in every eleven emerging adults will experience a major depressive episode each year (Mojtabai et al., 2016). Therefore, supporting the mental health of young people as they transition out of the adolescent period must be a global public health priority.

There is a growing body of literature demonstrating that healthy lifestyle behaviors including physical activity, screen time, sleep, and alcohol, tobacco and substance use are related to the prevention and treatment of mental health disorders (Firth et al., 2020; Groves et al., 2024). Adolescents who are more physically active (StrÖHle et al., 2007; Suetani et al., 2017) who do not smoke (Chaiton et al., 2009), and who sleep for longer periods of time each night (Orchard et al., 2020) are significantly less likely to have a mental health disorder in emerging adulthood. However, most of the research to date has focused on the association between individual healthy lifestyle behaviors and mental health. Yet, healthy lifestyle behaviors may cluster in unique ways (Leech et al., 2014; Meader et al., 2016), and therefore may have a multiplicative and synergistic effect on mental health outcomes. A recent meta-analysis did demonstrate that people who engage in healthier clusters of lifestyle behaviors report fewer symptoms of depression, anxiety, and general psychological distress, but notes a lack of longitudinal evidence (Bourke et al., 2025). There have been several cross-sectional studies which have demonstrated that clusters of healthy lifestyle behaviors relate to fewer symptoms of mental health conditions in young adults specifically (Brown et al., 2022; Champion et al., 2018; Di Benedetto et al., 2020; El Ansari et al., 2024; Kwan et al., 2016; Ye et al., 2016). Although there have been fewer longitudinal studies on the topic, results from longitudinal studies are consistent with the results reported in cross-sectional studies on the topic (Brown et al., 2021; Collins et al., 2023; Kleppang et al., 2023). However, a recent systematic review and meta-analysis of randomized controlled trials found inconsistent evidence of the effectiveness interventions which target multiple modifiable health behaviors on symptoms of depression and anxiety in adolescents and emerging adults (Bourke et al., 2022). Therefore, examining the nature and magnitude of the prospective association between patterns of healthy lifestyle behaviors during adolescence and emerging adulthood may provide insights into the extent to which, and potentially what type of, multi-faceted lifestyle interventions may be an effective approach to improving mental health outcomes during youth.

While it is important to consider the longitudinal association between the clustering of healthy lifestyle behaviors in adolescence with mental health outcomes in emerging adulthood, an equally important consideration is how lifestyle behaviors change during this dynamic life transition. Emerging adulthood is distinct from adolescence (Arnett, 2000). It is characterized by increased agency and independence, identity exploration, decreased social roles and obligations to others, and decreased social and institutional supports (Arnett, 2000; Arnett et al., 2014; Wood et al., 2018). This period of identity development and shifting interpersonal influences makes emerging adulthood a period of rapid changes in health behavior patterns (Nelson et al., 2008). Inauspiciously, there is consistent evidence that people tend to become unhealthier in emerging adulthood compared to adolescence (Frech, 2012; Harris et al., 2006; Paavola et al., 2004; Wiium et al., 2015). Specifically, emerging adults are more likely to engage in binge drinking, smoke, use illicit drugs, and be physically inactive when compared to their behaviors during adolescence. Therefore, the extent to which adolescents transition to less healthy clusters of lifestyle behaviors may be associated with mental health outcomes in emerging adulthood. Nevertheless, few studies have examined how clusters of health behaviors change longitudinally as individuals transition out of adolescence (Dakin et al., 2021; Lawrence et al., 2020), and to the authors knowledge, no studies have examined how changes in clusters of healthy lifestyle behaviors between adolescence and emerging adulthood are related to mental health outcomes.

The aim of this longitudinal study was to identify distinct latent profiles of healthy lifestyle behaviors during adolescence and emerging adulthood and examine the extent symptoms of psychological distress and mental wellbeing in young adulthood differs between the identified profiles. It is hypothesized that healthy combinations of lifestyle behaviors in adolescence will longitudinally relate to fewer symptoms of psychological distress and better mental wellbeing in emerging adulthood. Additionally, the study aims to determine how participants transition between profiles from adolescence to emerging adulthood and to investigate how different longitudinal transition patterns relate to psychological distress and mental wellbeing outcomes in young adulthood.

## Methods

### Procedures and Participants

Data for this study were from the Application of intergrateD Approaches to understanding Physical activity during the Transition to emerging adulthood (ADAPT) study (Kwan et al., 2020). This is a prospective cohort study which aimed to examine physical activity predictors and outcomes between the transition from adolescence (when participants were aged 15–18 years) to emerging adulthood (when participants were aged 18–21 years). Participants were recruited from a large school board located in Southern Ontario, Canada, and baseline data collection occurred in Fall, 2019. Adolescents attending a grade 11 class at baseline were eligible to participate in this study. From a total of 2412 adolescents eligible to participate in the study, 1585 responded to the invitation to participate, and 1238 had some valid data at baseline (51.3% valid response rate). Among the 1238 adolescents with valid data at baseline, 498 completed the follow-up assessment at three years post baseline (40.2% retention). Participants with missing data were excluded via listwise deletion, resulting in an analytic sample of 498 participants with valid data at both time points. A detailed description about the study procedures have been described previously (Kwan et al., 2020). The study procedures were approved by the Hamilton Integrated Research Ethics Board and the Hamilton-Wentworth Catholic District School Board.

### Measures

#### Independent Variables – Multiple Health Behaviors

##### Moderate-to-Vigorous Intensity Physical Activity

Moderate-to-vigorous intensity physical activity was assessed at baseline and follow-up using the International Physical Activity Questionnaire – Short Form (IPAQ-SF) (Craig et al., 2003). Participants were asked to report the number of days that they engaged in vigorous intensity and moderate intensity physical activity for at least 10 min at a time in the previous seven days. If they reported at least one day of moderate or vigorous physical activity, they were asked to report how much time they typically engaged in these activities on one of those days. Weekly time spent in moderate and vigorous physical activity was calculated as the number of days a participant was active multiplied by the amount of time they typically spent active on one of those days. Total time spent in moderate-to-vigorous intensity physical activity (MVPA) was calculated by adding the time spent in moderate and the time spent in vigorous physical activity in the previous seven days. Consistent with the IPAQ scoring guide, and to reduce the impact of outliers and remove infeasible values, total time spent in MVPA was truncated at 21 hr in the previous 7 days (i.e., 3 h per day).

##### Recreational Screen Time

Screen time was assessed at baseline and follow-up using a single item. Participants were asked to report the amount of time they spent watching TV or using a computer, tablet or smartphone during their free time on a typical day in the previous week. Participants were instructed not to include time on a computer at school or at work.

##### Sleep

Sleep duration was assessed at baseline and follow up. Participants responded to four items that assessed what time they typically went to sleep and woke up during weekdays and on the weekend over the past seven days. Responses were used to calculate the average number of hours participants slept on weekdays and weekends. Average daily sleep was then calculated by multiplying weekday sleep by five and weekend sleep by two, and then summing these products and dividing by seven.

##### Marijuana Use

Participants were asked to report on the number of occasions that they used marijuana (including a joint, pot, weed, hash, edibles) in the past 12 months at baseline and follow-up. Participants could select from nine response options ranging from never to every day. Reponses were collapsed into four categories: (1) not in the past 12 months, (2) less than once a month, (3) at least once a month, and (4) at least once a week.

##### Tobacco Smoking

Participants were asked to respond to two questions about tobacco smoking at baseline and follow-up. They were asked at the present time, how often they smoked cigarettes, and at the present time how often they used e-cigarettes of vapes. Response options were daily, occasionally, and not at all. Responses for cigarette smoking and vaping were combined to assess how often participants smoked tobacco.

##### Binge Drinking

Participants were asked to report on the number of occasions that they consumed five or more drinks on one occasion in the past 30 days at baseline and follow-up. They were instructed that one drink means one 341 ml or 12oz serving of beer whether from a bottle, can or draft, one 142 ml or 5oz glass of wine or bottle of cooler, or one straight or mixed drink with 1.5oz (43 ml) of liquor or spirit. Participants were asked to report on a six-point scale from never to daily or almost daily. Responses were collapsed into three categories: (1) not in the past month, (2) once in the past month, and (3) multiple times in the past month.

#### Outcomes

##### Psychological Distress

Symptoms of psychological distress was assessed at follow-up using Kessler’s Psychological Distress scale (K10; Kessler et al., 2002). It has demonstrated excellent convergent validity in large representative samples (Furukawa et al., 2003; Kessler et al., 2010) and among youth (Blake et al., 2023). Each of the ten items is scored on a five-point Likert with a score of one indicating that a symptom has not been present in the past 4 weeks and a score of five indicating a symptom has been present most of the time. Scores from each item are summed to calculate a total score ranging from 10 to 50.

##### Mental Wellbeing

Mental wellbeing was assessed at follow-up using the Warwick Edinburgh Mental Wellbeing Scale (WEMWBS; Tennant et al., 2007). The WEMWBS has strong psychometric properties in youth (McKay & Andretta, 2017). The WEMWBS assessed both the hedonic (i.e., happiness, satisfaction with life) and eudaimonic (i.e., self-realisation) aspects on mental wellbeing. Participants were asked to report how often they had experienced a range of positive aspects of mental wellbeing in the previous 2 weeks on a five-point scale from 1 (none of the time) to 5 (all of the time). The scale included 14 individual items, and the average item response was calculated as the overall scale score.

#### Covariates

Participant’s age at baseline, gender, and race were all recorded and included as covariates in the current study.

### Statistical Analysis

All statistical analysis was conducted using Mplus (v 8.4). Prior to analysis, the distribution of the independent variables was explored. The data for five participants were removed for having implausible outlier values for sleep (i.e., less than 3 hr or greater than 12 hr of sleep each night) at either baseline or follow-up.

The first step of the analysis involved estimating a cross-sectional latent profile model for the baseline data. The model was estimated with three continuous indicators (MVPA, screen time, sleep) and three categorical indicators (marijuana smoking, tobacco smoking, binge drinking). The model was estimated with a profile invariant, diagonal variance-covariance matrix, where the correlation between indicators within- profiles is fixed at zero and the variance of the indicators is fixed to be invariant across latent profiles. The best fitting model from profile enumeration was determined using the Bayesian Information Criteria (BIC; Gideon, 1978), which has been shown to perform well at identifying the correct number of latent profiles in a range of mixture modelling scenarios (Nylund et al., 2007). The entropy value was reported, which varies between zero and one, with a value closer to one indicating clearer delineation between the latent clusters (Celeux & Soromenho, 1996). Once the optimal number of latent profiles was identified, difference in symptoms of psychological distress and mental wellbeing between profiles was estimated using the manual three step approach (Vermunt, 2017). This approach fixes the conditional probabilities from modal class membership from the best-fitting unconditional model, meaning that additional distal outcomes can be estimated for each profile without influencing the estimation of class membership (Nylund-Gibson et al., 2019). Differences in psychological distress and mental wellbeing at follow-up between each of the profiles were estimated while controlling for participants gender, age, and race as covariates. In the second step, a latent profile analysis was conducted based on participants healthy lifestyle behaviours in emerging adulthood, with differences in psychological distress and mental wellbeing being subsequently examined.

In the third step, a latent transition analysis was estimated. Latent transition analysis is a mixture modelling technique which estimates the relationship between multiple latent profile variables longitudinally (Nylund-Gibson et al., 2023). Results from the latent profile analysis of follow-up data showed that there were substantive differences in the item means and profile-specific item probabilities compared to baseline, so invariance in the latent profiles across time was not imposed in the latent transition model. Instead, the model was estimated with the best fitting model from baseline and follow-up. The manual three-step approach was taken to estimate the latent transition model (Nylund-Gibson et al., 2014). Taking this approach means that the conditional probabilities of modal class membership at baseline and follow-up are fixed based on the results cross-sectional models, ensuring that the interpretation of the latent profiles remains consistent across cross-sectional and longitudinal analyses and meaning that class membership at baseline is not influenced by lifestyle behaviours at follow-up, ensuring temporal precedence. Additionally, using the manual three-step approach facilitates the inclusion of auxiliary variables without influencing the measurement model, and allows for the comparison of symptoms of psychological distress and mental wellbeing based on different latent transition patterns. Differences in psychological distress and mental wellbeing based on different latent transition patterns were assessed controlling for participant’s gender, and race as covariates. Standardized mean differences (i.e., Cohen’s d) are reported in text.

Given that latent profile and latent transition analysis are inherently exploratory (i.e., the number of latent profiles is not known before conducing the analysis), it is not feasible to conduct an a-priori power analysis. Nevertheless, a heuristic of ≥500 participants is generally suggested for latent profile and transition analyses (Nylund-Gibson & Choi, 2018; Nylund-Gibson et al., 2023), however, true power depends on several factors, such as the number of indicators included in the model and the degree of separation between each of the profiles (Tein et al., 2013). With regards to comparisons in distal outcomes between identified profiles and latent transition patterns, differences in outcomes were only reported for comparisons which had 80% power to identify at least a moderate-to-large difference between groups (i.e., d = 0.60). Remaining comparisons were considered to be unreliable, as the chance of detecting a true effect is reduced and the chance of overestimating an effect size is increased (Button et al., 2013).

## Results

### Participant Characteristics and Missing Value Analysis

Among the participants included in the analysis, most were female (68.2%), white (53.5%), and aged 16 years old at baseline (81.8%). Slightly more participants than not engaged in an average of 60 min of MVPA each day (52.4%), however, fewer slept between 8–10 hr each night (22.7%) or limited their recreational screen time to less than 2 hr per day (20.7%). The vast majority of participants had not consumed more than 5 alcoholic beverages on a single occasion in the past month (73.6%), had never used marijuana (75.1%), and had never smoked tobacco (81.9%) at baseline.

Participants with missing data at the follow-up assessment were significantly more physically active at baseline compared to participants with valid follow-up data (*p* < .001), and significantly younger (*p* = .017). Additionally, participants with missing data at follow-up were significantly more likely to binge drink at baseline (*p* = .002), and were significantly more likely to be male (*p* < .001). Missing data was not related to participants levels of recreational screen time or sleep at baseline, whether participants smoked marijuana or tobacco at baseline, or participant’s race.

### Baseline Latent Profile Analysis

The model fit statistics from the estimation of the mixture model at baseline with increasing numbers of estimated latent profiles in displayed in Appendix A. Results showed that the 3-profile solution was the best fitting model. Lifestyle behaviors for each of the latent profiles are displayed in Table 1. The majority of participants were assigned to profile 1 (74.3%) which was characterized as moderately active abstainers. Fewer participants were assigned to profile 2 (16.9%), which were high screen time users, or profile 3 (8.7%) which were characterized as moderately active with moderate risk behaviors.

**Table 1.** Lifestyle Behaviors for each Latent Profile at Baseline

	Profile 1	Profile 2	Profile 3
	“Moderately active abstainers” (*n* = 366)	“High screen time users” (*n* = 83)	“Moderately active with moderate risk behaviors” (*n* = 43)
MVPA hrs/week	8.39 (7.88, 8.90)	7.15 (5.56, 8.74)	9.45 (7.98, 10.92)
Screen time hrs/day	4.02 (3.75, 4.29)	14.54 (13.58, 15.50)	4.13 (2.46, 3.80)
Sleep	7.34 (7.25, 7.43)	7.18 (6.74, 7.62)	7.09 (6.90, 7.28)
Marijuana			
Not in the past 12 months	98.4%	81.8%	14.5%
Less than once a month	1.6%	7.4%	31.7%
At least once a month	0.0%	6.4%	30.6%
At least once a week	0.0%	4.4%	23.2%
Tobacco			
Never	96.7%	75.6%	28.8%
Occasionally	3.3%	12.7%	47.5%
Daily	0.0%	11.6%	23.7%
Binge drinking			
Not in the past month	90.3%	79.3%	32.3%
Once in the past month	8.2%	2.2%	35.4%
Multiple times in the past month	1.6%	18.5%	32.3%

*Note.* MVPA = moderate-to-vigorous physical activity.

The longitudinal association between profile membership in adolescence and psychological distress and mental wellbeing during emerging adulthood are displayed in Table 2. Results demonstrated that the moderately active abstainers reported significantly fewer symptoms of psychological distress compared to participants who were in the moderately active with moderate risk behaviors profile as adolescents (d = −0.42, *p* = .001). Additionally, the moderately active abstainers reported significantly more positive mental wellbeing compared to adolescents in the high screen time use profile as adolescents (d = 0.90, *p* < .001).

**Table 2.** Differences in Psychological Distress (K10) and Mental Wellbeing (WEMWBS) at Follow-up Between Profiles at Baseline

K10				
	Mean	Profile 1	Profile 2	Profile 3
Profile 1	24.32 (23.60, 25.04)	^-^	−1.63 (−3.89, 0.63)	−2.70 (−4.15, −1.24)**
Profile 2	25.95 (24.10, 27.79)		-	−1.07 (−4.12, 1.98)
Profile 3	27.01 (24.96, 29.06)			-

WEMWBS				
	Mean	Profile 1	Profile 2	Profile 3
Profile 1	48.27 (46.20, 50.34)	-	3.41 (2.24, 4.58)**	2.43 (−0.65, 5.50)
Profile 2	44.86 (42.19, 47.53)		-	−0.98 (−3.35, 1.38)
Profile 3	45.84 (42.40, 49.28)			-

*Note.* Results are presented as mean (95% confidence interval); *p < .01 **p < .001, WEMWBS = warwick edinburgh mental wellbeing scale.

### Follow-Up Latent Profile Analysis

Model fit statistics for the latent profile analysis at follow-up are displayed in Appendix A. Although results demonstrated improving model fit with increasing number of profiles, there were small profiles which consisted of less than 5% of the total study population for models which estimated more than four latent profiles, indicating potentially spurious profiles. Therefore, the four-profile solution was chosen as the optimal solution at follow-up. Lifestyle behaviors for each of the profiles at follow-up are displayed in Table 3. Participants were relatively evenly split between the four profiles which included Profile 1 (23.4%) which was characterized by highly active with moderate risk behaviors, Profile 2 (23.6%) which was characterized by moderately active with moderate risk, Profile 3 (31.3%) which was characterized by inactive abstainers, and Profile 4 (21.7%) which was characterized by inactive with risky behaviors. Sleep duration was relatively consistent across the profiles.

**Table 3.** Lifestyle Behaviors for each Latent Profile at Follow-up

	Profile 1	Profile 2	Profile 3	Profile 4
	“Highly active with moderate risk behaviors” (*n* = 115)	“Moderately active with moderate risk behaviors” (*n* = 116)	“Inactive abstainers” (*n* = 154)	“Inactive with risky behaviors” (*n* = 107)
MVPA hrs/week	19.99 (19.60, 20.38)	10.74 (9.66, 11.82)	2.13 (0.66, 3.60)	2.89 (2.15, 3.63)
Screen time hrs/day	5.32 (4.67, 5.97)	4.84 (4.11, 5.57)	5.30 (4.63, 5.97)	5.00 (4.24, 5.76)
Sleep	6.89 (6.52, 7.26)	7.18 (6.83, 7.53)	7.11 (6.91, 7.31)	7.99 (7.79, 8.19)
Marijuana				
Not in the past 12 months	51.8%	53.7%	86.5%	1.7%
Less than once a month	14.9%	11.6%	12.6%	23.9%
At least once a month	15.3%	15.1%	0.9%	29.3%
At least once a week	17.9%	19.6%	0.0%	45.1%
Tobacco				
Never	71.6%	78.9%	97.8%	48.0%
Occasionally	12.0%	12.6%	1.1%	29.1%
Daily	16.4%	8.5%	1.1%	22.9%
Binge drinking				
Not in the past month	32.2%	38.0%	70.3%	13.0%
Once in the past month	22.9%	17.0%	17.8%	12.9%
Multiple times in the past month	44.9%	45.0%	11.8%	74.1%

*Note.* MVPA = moderate-to-vigorous physical activity.

Differences in symptoms of psychological distress and mental wellbeing between each of the identified profiles at follow-up are displayed in Table 4. Results indicate that there were no significant differences in symptoms of distress or levels of mental wellbeing between any of the profiles at follow-up.

**Table 4.** Differences in Psychological Distress (K10) and Mental Wellbeing (WEMWBS) Between Profiles at Follow-up

K10					
	Mean	Profile 1	Profile 2	Profile 3	Profile 4
Profile 1	24.93 (24.09, 25.77)	^-^	0.88 (−0.99, 2.75)	0.14 (−1.03, 1.30)	−1.37 (−4.18, 1.44)
Profile 2	24.05 (21.99, 26.10)		-	−0.74 (−2.84, 1.35)	−2.25 (−5.97, 1.47)
Profile 3	24.79 (23.72, 25.86)			-	−1.50 (−3.70, 0.70)
Profile 4	26.29 (24.02, 28.56)				-

WEMWBS					
	Mean	Profile 1	Profile 2	Profile 3	Profile 4
Profile 1	48.94 (46.09, 51.80)	-	1.58 (−1.03, 4.18)	2.57 (−0.27, 5.42)	2.77 (−1.31, 6.85)
Profile 2	47.37 (44.89, 49.84)		-	1.00 (−1.27, 3.26)	1.19 (−2.27, 4.65)
Profile 3	46.37 (43.93, 48.81)			-	0.20 (−1.85, 2.2)
Profile 4	46.17 (42.12, 50.22)				-

*Note.* Results are presented as mean (95% confidence interval), WEMWBS = warwick edinburgh mental wellbeing scale.

### Latent Transition Analysis

**Figure 1.**
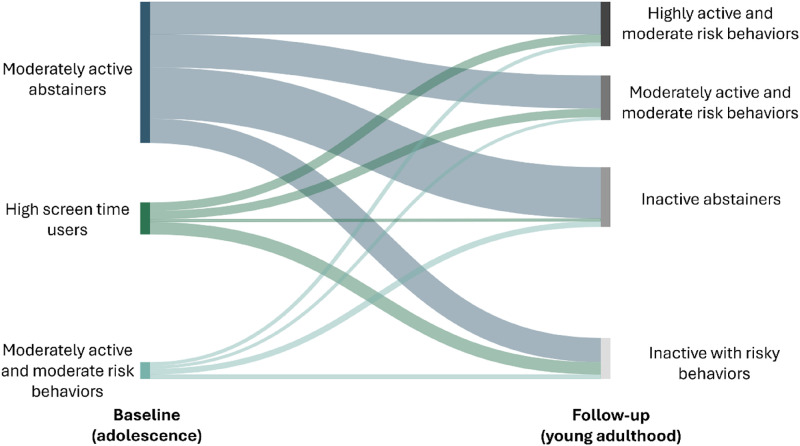
Transition Between Classes From Adolescents to Emerging Adulthood

Results from the latent transition analysis are displayed in Figure 1. Participants in Profile 1 (moderately active abstainers) at baseline most commonly transitioned to Profile 3 (inactive abstainers) at follow-up (36.3%), followed by Profile 2 (moderately active with moderate risk behaviors; 23.5%), Profile 1 (highly active with moderate risk behaviors; 23.0%) and Profile 4 (inactive with risky behaviors; 17.2%). Participants in Profile 2 (high screen time users) at baseline were most likely to transition to Profile 4 at follow-up (inactive with risky behaviors; 38.6%) followed by Profile 1 or 2 (moderate or highly active with moderate risk behaviors; both 26.5%), and Profile 3 (inactive abstainers; 8.4%). Participants in Profile 3 (moderately active with moderate risk behaviors) at baseline were most likely to transition into Profile 3 (inactive abstainers; 34.9%) followed by Profile 4 (inactive with risky behaviors; 25.6%), Profile 1 (highly active with moderate risk behaviors; 20.9%) and Profile 2 (moderately active with moderate risk behaviors; 18.6%).

Differences in symptoms of psychological distress and mental wellbeing between participants with different latent transition patterns are displayed in Table 5. There were small cell sizes of participants who transitioned from Profiles 2 (high screen time users) and 3 (moderately active with moderate risk behaviors) at baseline to various profiles at follow-up, thus these results may not be reliable. Therefore, only results are for participants transitioning from Profile 1 (moderately active abstainers) at baseline to various profiles at follow-up are presented. Results demonstrated that there was no significant difference in symptoms of psychological distress of mental wellbeing between participants who transitioned from Profile 1 (moderately active abstainers) at baseline to various profiles at follow-up.

**Table 5.** Differences in Psychological Distress (K10) and Mental Wellbeing (WEMWBS) Among Participants who Transitioned From Profile 1 at Baseline to Various Profiles at Follow-up

K10					
Transitioned to	Mean	Profile 1	Profile 2	Profile 3	Profile 4
Profile 1	23.47 (20.99, 25.94)	^-^	−0.16 (−2.47, 2.15)	−1.47 (−3.77, 0.83)	−2.30 (−7.85, 3.25)
Profile 2	23.63 (21.70, 25.56)		-	−1.31 (−3.59, 0.97)	−2.14 (−6.79, 2.52)
Profile 3	24.94 (23.91, 25.97)			-	−0.82 (−4.29, 2.64)
Profile 4	25.76 (22.57, 28.96)				-

WEMWBS					
Transitioned to	Mean	Profile 1	Profile 2	Profile 3	Profile 4
Profile 1	49.25 (45.28, 53.22)	-	1.51 (−2.23, 5.26)	2.28 (−2.94, 7.51)	1.14 (−6.46, 8.74)
Profile 2	47.74 (44.94, 50.52)		-	0.77 (−2.28, 3.82)	−0.37 (−5.42, 4.68)
Profile 3	46.97 (44.47, 49.47)			-	−1.14 (−3.73, 1.45)
Profile 4	48.11 (43.30, 52.92)				-

*Note.* Results are presented as mean (95% confidence interval), WEMWBS = warwick edinburgh mental wellbeing scale.

## Discussion

The aim of this study was to examine how multiple lifestyle behaviors during youth were related to symptoms of psychological distress and mental wellbeing in emerging adulthood. Results demonstrated that adolescents with the healthiest combinations of lifestyle behaviors reported significantly better mental health in emerging adulthood. Specifically, participants who were moderately active abstainers in adolescence reported fewer symptoms of psychological distress when compared to participants who were moderately active but engaged in moderately high levels of risky behaviors during adolescence. Additionally, moderately active abstainers reported significantly higher levels of mental wellbeing in emerging adulthood when compared to adolescents who were high screen time users. Interestingly, lifestyle behaviors during emerging adulthood were not associated with mental health and wellbeing in emerging adulthood, nor were there difference in mental health outcomes among participants who were moderately active abstainers as an adolescent who transitioned into various profiles of healthy lifestyle behaviors during emerging adulthood.

The results from the current study support the hypothesis that engaging in healthy lifestyle behaviors as an adolescent can protect against developing symptoms of psychological distress and promote positive mental wellbeing in emerging adulthood. With regards to psychological distress, the finding that participants who were moderately active but also engaged in moderate levels of risky behaviors as adolescents reported greater symptoms of psychological distress in emerging adulthood compared to participants who were moderately active abstainers as adolescents is consistent with previous literature which has demonstrated a prospective relationship between alcohol use, smoking, and marijuana use in adolescents with symptoms of distress in emerging adulthood (Brook et al., 2002; Gobbi et al., 2019; Wells et al., 2004). Adolescence is a period of heightened neuroplasticity, and is a sensitive period of brain development (Fuhrmann et al., 2015). Binge drinking, tobacco smoking, and marijuana use may impact the architecture and functioning of adolescent’s brains, including regions implicit in reward processing and executive functioning (Blest-Hopley et al., 2018; Lydon et al., 2014; Pérez-García et al., 2022); regions of the brain which may also be altered in individual’s with depression (Drevets et al., 2008; Zhang et al., 2018). Therefore, increasing the age of onset of binge drinking and marijuana use, and reducing the number of adolescents who smoke tobacco may reduce the incidences of mental health disorders as youth transition beyond secondary school.

With regards to mental wellbeing, children who were high screen users in adolescence reported significantly worse mental wellbeing in emerging adulthood. The existing research on the longitudinal association between screen time and mental health and wellbeing has been somewhat mixed (Tang et al., 2021). This could potentially be a result of the fact that different types of screen time may be differently associated with mental health outcomes in young people (Sanders et al., 2024). For example, cognitively active screen activities may actually be beneficial for mental health (Hallgren et al., 2020). However, evidence demonstrates that excessive screen use is unfavorably related to several aspects of wellbeing in young people including satisfaction with life and loneliness (Khan et al., 2021; Lawrence et al., 2022). Not all screen time is intrinsically harmful. For example, mentally active screen time, such as playing video games, may not be as detrimental to mental health as mentally passive sedentary behaviors in emerging adults (Peng et al., 2024). However, the high levels of screen time observed among the high screen time users in the current study, the impact on mental wellbeing is detrimental (Przybylski & Weinstein, 2017). As smartphones have become ubiquitous in developed societies, even among youths, it is not surprising that the prevalence of smartphone addiction has increased (Olson et al., 2022), which might contribute to very high levels of screen time observed in the current study, especially considering that the majority of the sample were girls (Leonhardt & Overå, 2021). Intervening in young people with problematic smartphone use patterns may be essential to promote positive aspects of mental wellbeing in adolescents and emerging adults (White et al., 2024).

Unlike adolescence, healthy lifestyle behaviors during emerging adulthood were not significantly related to symptoms of psychological distress or mental wellbeing. Additionally, the changes in profiles among those who were in the moderately healthy abstainers were not related to symptoms of psychological distress or mental wellbeing. One potential explanation for this result is that, although there was a clear profile which had the unhealthiest lifestyle behaviors, there was not a consistently salubrious profile for emerging adults. Those who abstained from alcohol, tobacco, and marijuana were relatively inactive, while emerging adults who were highly active were likely to binge drink, and to a lesser extent smoke tobacco and use marijuana. This is consistent with cross-sectional research which has demonstrated a positive correlation between physical activity and alcohol consumption in this population (Dodge et al., 2016). However, the association between physical activity and binge drinking may be less pronounced in young women (Barry et al., 2013; Buscemi et al., 2011), meaning that the number of emerging adults assigned to the moderately active with moderate risk behaviors profile may be larger in a more gender balanced sample. The results from the current study are similar to results that have been reported in previous studies examining the clustering of healthy lifestyle behaviors in emerging adults, which have found that the healthiest cluster only exhibit moderately healthy lifestyle behaviors (Collins et al., 2023; Kwan et al., 2016) or that few emerging adults engage in consistently healthy behaviors (Lawrence et al., 2017). This highlights how, contrary to popular belief, emerging adults are surprisingly unhealthy (Stroud et al., 2015). Additionally, there was no clear pattern between adolescents who engage in healthy lifestyle behaviors transitioning to any particular latent profile at follow-up. This highlights how emerging adulthood is a distinct life period from adolescence, where an individual has increased independence, decreased social and institutional obligations and supports, and participates in identity exploration, which may leave them susceptible to engaging in risky and unhealthy behaviors (Arnett, 2000; Arnett et al., 2014; Nelson et al., 2008; Wood et al., 2018). Therefore, supporting youth to continue to engage in healthy lifestyle behaviors prior to and after the transition beyond high school may be necessary to ensure favorable outcomes in this crucial developmental period of youth and is a growing public health priority. Given that multiple behavior change interventions may be more effective (Zhang et al., 2024), taking a holistic human-centered approach to improving lifestyle behaviors where the largest benefits can be gained for each individual may be effective in this cohort (Bourke et al., 2025). Nevertheless, more research is needed to determine how best to support an overall healthy combination of lifestyle behaviors among emerging adults.

### Limitations

This study had several strengths, including the use of a person-centered analysis to examine the conjoint longitudinal association between lifestyle behaviors and indicators of mental health in emerging adults. Nevertheless, there are several limitations which must be acknowledged. Firstly, all data in the current study was self-reported. Despite the use of valid and reliable instruments, there are several biases which may be introduced when examining self-reported data. These include recall bias, social desirability bias, and common method bias (although this is less of a concern when examining longitudinal data). Future studies could benefit from the use of device-assessed physical activity, sedentary behavior, and sleep (e.g., accelerometry) and higher resolution documenting of health-risk behaviors such as binge drinking, tobacco smoking, and marijuana use. Additionally, using more objective assessments for mental health, such as diagnostic interviews, may strengthen the results of future research. Second, there was some inconsistencies in the temporal frame that lifestyle behaviors were being assessed. For example, physical activity and screen time were assessed in the previous seven days, current smoking and vaping behaviors were assessed, marijuana use was assessed over the participants lifetime, and binge drinking was assessed over the past month. Another limitation of the current study was that psychological distress and mental wellbeing were not assessed at baseline, so changes in our outcomes of interest could not be assessed, meaning that reverse causation cannot be ruled out. Additionally, important socio-demographic data which may have confounded the results were not available (e.g., socioeconomic status indicators) and therefore could not be controlled for in the present analysis. Finally, attrition analysis demonstrated that data at follow-up was not missing completely at random. Specifically, participants with missing data were more active and more likely to binge drink at baseline, indicating that they may have been more likely to fall into the moderately active with moderate risk behaviors profile at baseline. Therefore, the number of participants in the moderately active with moderate risk behaviors profile may have been underestimated in the present analysis. Although statistical methods such as Full Information Maximum Likelihood (FIML) and multiple imputation were considered, they could not be implemented. For example, FIML cannot handle cases with missing data on all indicator variables (such is the case when a participant is lost to follow-up), while multiple imputation may lead to different optimal class solutions for each imputed dataset. This limitation should be taken into account when interpreting the profile distribution.

## Conclusion

Results from this study demonstrate that engaging in more healthy lifestyle behaviors in adolescence may protect against psychological distress and promote mental wellbeing. Neither clusters of lifestyle behaviors in emerging adulthood, nor transitions in lifestyle behaviors from adolescence to emerging adulthood, were related to mental health outcomes. Nevertheless, results demonstrated that most participants transitioned to less healthy lifestyle behaviors in emerging adulthood, so it may be crucial to support the maintenance of a healthy lifestyle in this important transition period.

## Appendix A

### Model Fit Statistics from the Latent Profile Analysis at Baseline and Follow-up

**Table A1.** Model Fit Statistics From the Baseline Latent Profile Analysis

	BIC	saBIC	Entropy	Smallest profile
2 profiles	9006.26	8930.08	.877	18.7%
3 profiles	8859.54	8748.45	.902	8.7%
4 profiles	8885.88	8739.88	.921	1.2%
5 profiles	8909.78	8728.86	.908	1.4%
6 profiles	8928.69	8712.86	.868	2.0%

*Note.* BIC = bayesian information criteria; saBIC = sample-size adjusted bayesian information criteria.

**Table A2.** Model Fit Statistics for the Latent Profile Analysis at Follow-up

	BIC	saBIC	Entropy	Smallest
2 profiles	10,317.66	10241.48	.740	43.5%
3 profiles	10,218.12	10,107.03	.856	28.5%
4 profiles	10,195.26	10049.29	.878	21.7%
5 profiles	10,191.29	10010.37	.890	3.0%

*Note.* BIC = bayesian information criteria; saBIC = sample-size adjusted bayesian information criteria.
